# Study of EEG sensitivity and specificity in loss of conciousness in adolescents

**DOI:** 10.1192/j.eurpsy.2024.924

**Published:** 2024-08-27

**Authors:** R. Bouchech, A. Fki, I. Kammoun, I. Kammoun, K. Masmoudi

**Affiliations:** ^1^Functional explorations department, Habib Bourguiba hospital, Sfax; ^2^Occupational medicine department, Sahloul university hospital, Sousse; ^3^functional explorations department, Hedi chaker hospital, Sfax, Tunisia

## Abstract

**Introduction:**

Although the etiological diagnosis of loss of consciousness is essentially based on a careful history and clinical examination, electroencephalography (EEG) remains an important investigative tool.

**Objectives:**

The aim of this study was to identify the value of EEG in the management of adolescents with recurrent bouts of fainting

**Methods:**

This was a retrospective descriptive study conducted from January 2019 to May 2022. We included all adolescents referred to the functional explorations department at Habib Bourguiba hospital, Tunisia for Electroencephalogram (EEG) as part of a workup to explore recurrent episodes of loss of consciousness.

**Results:**

A total of 55 adolescents were included in this study, with a mean age of 15.4±2.3 and a 72.4% female proportion.The delay between the EEG and the onset of the seizure was greater than one week.67.3% of patients were referred by the child psychiatry department.29.1% of the 55 EEG reports were pathological. Epileptiform discharges were noted in 56.3% of adolescents. Slow waves were found in 43.7% of cases. The location of the abnormalities was predominantly frontal. Patients with temporal EEG anomalies had a notion of ascending epigastric pain preceding loss of consciousness in 90% of cases. Adolescents with EEGs containing epileptiform abnormalities had a history of paroxysmal movements in 30% of cases. The sensitivity of the EEG was estimated to be around 25%, and the specificity around 79%.

**Conclusions:**

Although the clinical examination is of great importance in the etiological diagnosis of loss of consciousness, the EEG remains a complementary examination of non-negligible interest in the etiological investigation.

**Disclosure of Interest:**

None Declared

